# Insecticidal Toxicity of *Yersinia frederiksenii* Involves the Novel Enterotoxin YacT

**DOI:** 10.3389/fcimb.2018.00392

**Published:** 2018-11-14

**Authors:** Katharina Springer, Philipp-Albert Sänger, Christian Moritz, Angela Felsl, Thomas Rattei, Thilo M. Fuchs

**Affiliations:** ^1^Lehrstuhl für Mikrobielle Ökologie, Fakultät für Grundlagen der Biowissenschaften, Wissenschaftszentrum Weihenstephan, Technische Universität München, Freising, Germany; ^2^Friedrich-Loeffler-Institut, Institut für Molekulare Pathogenese, Jena, Germany; ^3^Department of Computational Systems Biology, University of Vienna, Vienna, Austria

**Keywords:** *Yersinia*, *Galleria mellonella*, insecticidal activity, enterotoxin, YacT

## Abstract

The genus *Yersinia* comprises 19 species of which three are known as human and animal pathogens. Some species display toxicity toward invertebrates using the so-called toxin complex (TC) and/or determinants that are not yet known. Recent studies showed a remarkable variability of insecticidal activities when representatives of different *Yersinia* species (spp.) were subcutaneously injected into the greater wax moth, *Galleria mellonella*. Here, we demonstrate that *Y. intermedia* and *Y. frederiksenii* are highly toxic to this insect. A member of *Y. Enterocolitica* phylogroup 1B killed *G. mellonella* larvae with injection doses of approximately 38 cells only, thus resembling the insecticidal activity of *Photorhabdus luminescens*. The pathogenicity *Yersinia* spp. displays toward the larvae was higher at 15°C than at 30°C and independent of the TC. However, upon subtraction of all genes of the low-pathogenic *Y. enterocolitica* strain W22703 from the genomes of *Y. intermedia* and *Y. frederiksenii*, we identified a set of genes that may be responsible for the toxicity of these two species. Indeed, a mutant of *Y. frederiksenii* lacking *yacT*, a gene that encodes a protein similar to the heat-stable cytotonic enterotoxin (Ast) of *Aeromonas hydrophila*, exhibited a reduced pathogenicity toward *G. mellonella* larvae and altered the morphology of hemocytes. The data suggests that the repertoire of virulence determinants present in environmental *Yersinia* species remains to be elucidated.

## Introduction

The genus *Yersinia* so far consists of three human pathogens (*Y. pestis, Y. pseudotuberculosis*, and *Y. enterocolitica*), and at least 16 species are considered mostly harmless to humans, namely *Y. aldovae, Y. bercovieri, Y. frederiksenii, Y. intermedia, Y. kristensenii, Y. mollaretii, Y. rohdei*, and the more recently described *Y. ruckeri*, a fish pathogen (Sulakvelidze, 2000), *Y. aleksiciae* (Sprague and Neubauer, 2005), *Y. similis* (Sprague et al., 2008), *Y. massiliensis* (Merhej et al., 2008), *Y. nurmii* (Murros-Kontiainen et al., 2010a), *Y. pekkanenii* (Murros-Kontiainen et al., 2010b), *Y. wautersii* (Savin et al., 2014), and *Y. entomophaga* (Hurst et al., 2010). These species that are non-pathogenic for humans have been isolated from water, soil, food, domestic and wild animals, and human beings in which they do not cause any clinical infections.

While the virulence properties of the pathogenic species have been characterized during last few decades, much less is known about the determinants that allow *Yersinia* species to survive in the environment. We have recently demonstrated that the *Y. enterocolitica* strain W22703 (biotype 2, serotype O: 9) is toxic to nematodes and larvae of the tobacco hornworm, *Manduca sexta*, upon oral infection or oral toxin application, and that this insecticidal activity correlates with the presence of the pathogenicity island (TC-PAI^*Ye*^) (Bresolin et al., 2006; Spanier et al., 2010). This 20-kb fragment is present in the genome of *Y. pestis, Y. pseudotuberculosis*, and *Y. enterocolitica* biotype 2–5 strains, but is absent in the genomes of the highly—pathogenic biotype 1B strains, including 8081 and of the most biotype 1A strains, which are considered to be non-pathogenic to humans. The TC-PAI^*Ye*^ carries the toxin complex (TC) genes with high identity to the *tc* genes of entomophagous *Photorhabdus luminescens*, and it might be speculated that the TC is required to penetrate the epithelial cell barrier of the insect gut to allow *Y. enterocolitica* cells to enter the hemocoel. In *Y. entomophaga* MH96, an insecticidal pathogenicity island termed PAI_Ye96_ was characterized, which is distinct from TC-PAI^*Ye*^ in terms of gene homology and genetic organization (Hurst et al., 2011). A unique feature of this island is that it encodes, besides the type ABC genes, two chitinases that are associated with the mature TC (Busby et al., 2012).

The transcription of the *tc* genes in *Y. enterocolitica* is subject to a strict temperature-dependent regulation as they are completely silenced at 37°C, but strongly upregulated at lower temperatures with a maximal transcription at 10–15°C approximately (Bresolin et al., 2006; Starke et al., 2013; Starke and Fuchs, 2014). Thus, the TC-dependent activity of *Y. enterocolitica* to invertebrates is reciprocally regulated in comparison with that of many *Yersinia* virulence factors directed against humans (Marceau, 2005). Notably, strains lacking the insecticidal genes, including *Y. enterocolitica, Y. mollaretii, Y. bercovieri, Y. ruckeri*, and *Y. aldovae*, are still toxic when subcutaneously injected into *G. mellonella*, indicating the presence of yet unknown insecticidal determinants in these species (Fuchs et al., 2008). More recently, it was demonstrated that *Y. enterocolitica* strains of phylogroup 1 exhibit a strong virulence against *G*. *mellonella* larvae at temperatures of 25°C and higher upon intrahemocoelic injection, independently of the presence of virulence plasmid pYV (Alenizi et al., 2016). These findings resemble functional redundancy in *P. luminescens* that carries a set of insecticidal factors besides the TC (ffrench-Constant et al., 2007), including the *makes caterpillars floppy* (MCF) toxins (Daborn et al., 2002), the Pir toxins (Waterfield et al., 2005), the protease PrtA (Bishop, 2014), Txp40 (Brown et al., 2006), the XaxAB-like binary toxins (Zhang et al., 2014), and the *Photorhabdus* virulence cassettes (Yang et al., 2006). Interestingly, the (partial) loss of some of these insecticidal genes does not result in a lack of toxicity to invertebrates (Wilkinson et al., 2009).

The insecticidal determinants of some *Yersinia* spp. remain to be investigated. Here, we used an established infection assay with *G. mellonella* larvae to monitor the phenotype of the host intrahemocoelically infected with *Y. frederiksenii, Y. intermedia, P. luminescens*, and *Y. enterocolitica* 8081 and W22703 cells. Dose- and temperature-dependent characteristics of their insecticidal activity were determined, and genome comparison as well as toxin injection were applied to gain further insights into the entomopathogenic repertoire of environmental *Yersinia* strains.

## Results

**Figure 1:**
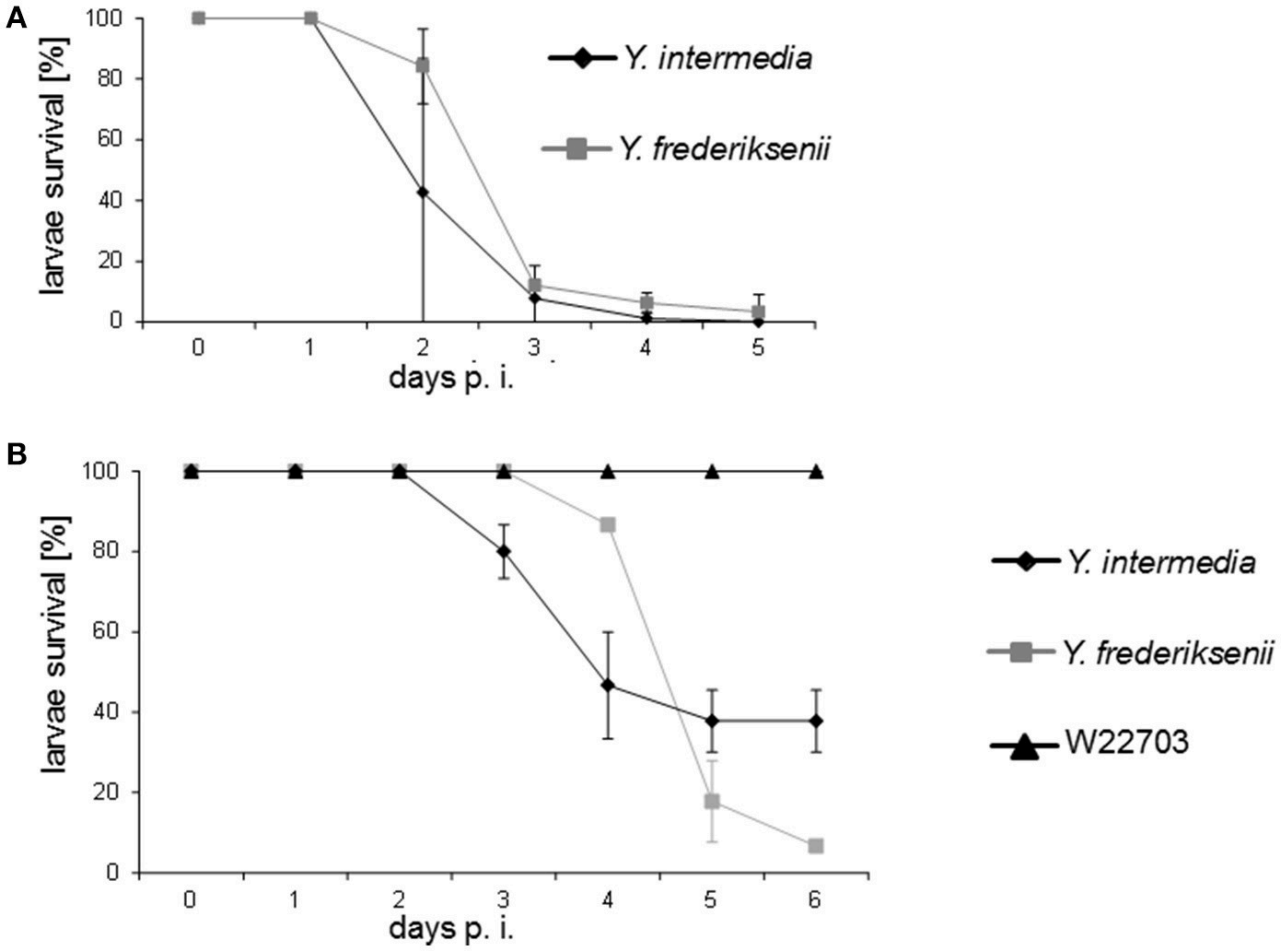
Time course of *G. mellonella* infection assays with *Y. frederiksenii* and *Y. intermedia*. The strains were pregrown overnight at 30°C, and the cultures were serially diluted. Aliquots of 5–7.5 μl from a 10^4^-fold or a 10^5^-fold dilution were used for infection, corresponding to **(A)** 1.44–5.50 × 10^3^ *Y. intermedia* CFU and 1.43 × 10^3^–10^4^ *Y. frederiksenii* CFU, or **(B)** 120 *Y. intermedia* CFU, 95 *Y. frederiksenii* CFU, and 145 *Y. enterocolitica* W22703 CFU. Three independent experiments per strain were performed, with three groups composed of **(A)** 25, 29, and 30 larvae (*Y. intermedia*) and 20, 22, and 23 larvae (*Y. frederiksenii*), or **(B)** 15 larvae each. The larvae were incubated at 20°C and monitored daily. Error bars represent the standard error of the mean of three experiments.

Dose-dependent toxicity of *Y. frederiksenii* and *Y. intermedia* to *G. mellonella* larvae. When larvae of the first-instar neonates of *M. sexta* were challenged orally with *Y. frederiksenii* and *Y. intermedia* in preliminary experiments, the toxicity was calculated as 71 and 19%, respectively (Table 1). As both strains used here lack the TC-PAI^*Ye*^, we switched from this oral infection model to a subcutaneous infection model and injected 5 μl of a 1:100 dilution of *Y. frederiksenii* and *Y. intermedia* overnight culture into *G. mellonella* larvae, and observed a 99–100% lethality after 5 days of incubation at 15°C (Table 1). Thus, the insecticidal activity of *Y. frederiksenii* and *Y. intermedia* is higher than that of all other *Yersinia* strains tested recently in the same infection model, including *Y. enterocolitica* strain W22703 (biotype 2, serotype O:9) (Fuchs et al., 2008). To determine the toxicity of *Y. intermedia* and *Y. frederiksenii* toward *G. mellonella* larvae in more detail, we used defined infection aliquots of approximately 10^3^–10^4^ colony forming units (CFU), and monitored the fate of intrahemocoelically infected *G. mellonella* larvae over 5 days at room temperature (20°C) (Figure 1A). The timecourse revealed that all larvae survived for 24 h, but most of them died within the next 2 days. The survival rates of the animals infected with two *Yersinia* species did not significantly (*p* > 0.05) differ under the conditions applied here. In parallel, we homogenized two larvae each day, and monitored the replication of *Y. frederiksenii* within the larvae. The CFU of *Y. frederiksenii* increased from 1.15 × 10^3^ to 3.21 × 10^3^ (day one) and to 2.79 × 10^9^ (day two) directly after the infection, and remained constant for next 3 days (1.88–2.62 × 10^9^). This unimpeded bacterial growth resembles the mortality of the larvae that starts only when the *Y. frederiksenii* reaches its stationary phase. This finding suggests that a high cell number of this insect pathogen in the larvae of *Galleria* is a prerequisite for its toxicity and/or that the pathogen has incapacitated the host in the initial phase of intrahemocoelical infection.

**Table 1:** Oral infection of *M. sexta* and intrahemocoelic infection of *G. mellonella* for 5 days.

**Infection model**	**Strain**	***tc-*PAI*^*Ye*^***	**Total no**.	**Dead**	**Alive**	**Dead**	**Alive**	**Dead**	**Alive**	**Dead [%] ± Sd^a^**
*G. mellonella*				1:10		1:100		total		
	*Y. intermedia*	Absent^b^	52	25	0	27	0	52	0	**100** ± **0**
	*Y. frederiksenii*	Plasmid-encoded *sep*-like genes absent in strain CIP 80.29^c^	68	28	0	39	1	67	1	**99** ± **2**
	**Controls**									
	*E. coli* DH5α	Absent^b^	63	5	34	2	22	7	56	**13** ± **6**
	LB		64					3	61	**5** ± **0**
*M. sexta*			Undiluted							
	*Y. intermedia*	Absent^b^	27	5	22					**19** ± **14**
	*Y. frederiksenii*	Plasmid-encoded *sep*-like genes absent in strain CIP 80.29^c^	21	15	6					**71** ± **4**
	**Control**									
	DH5α	Absent^b^	21	1	20					**5** ± **7**

a The average mortality of at least three independently performed experiments with a minimum of six larvae each are shown

b according to the genome sequence

C *(Fuchs et al., 2008)*.

We further reduced the infection dose to approximately 100–150 CFU and observed a slightly higher toxicity of *Y. frederiksenii* in comparison with *Y. intermedia*, which, however, started to kill larvae a day earlier (Figure 1B). In contrast, 145 CFU of *Y. enterocolitica* W22703 were not sufficient to kill any larva. Altogether, these data demonstrate a high, dose-dependent toxicity of *Y. frederiksenii* and *Y. intermedia* toward larvae of *G. mellonella*.

### Entomopathogenicity of *Y. enterocolitica* strain 8081 resembles that of *P. luminescens*

**Figure 2:**
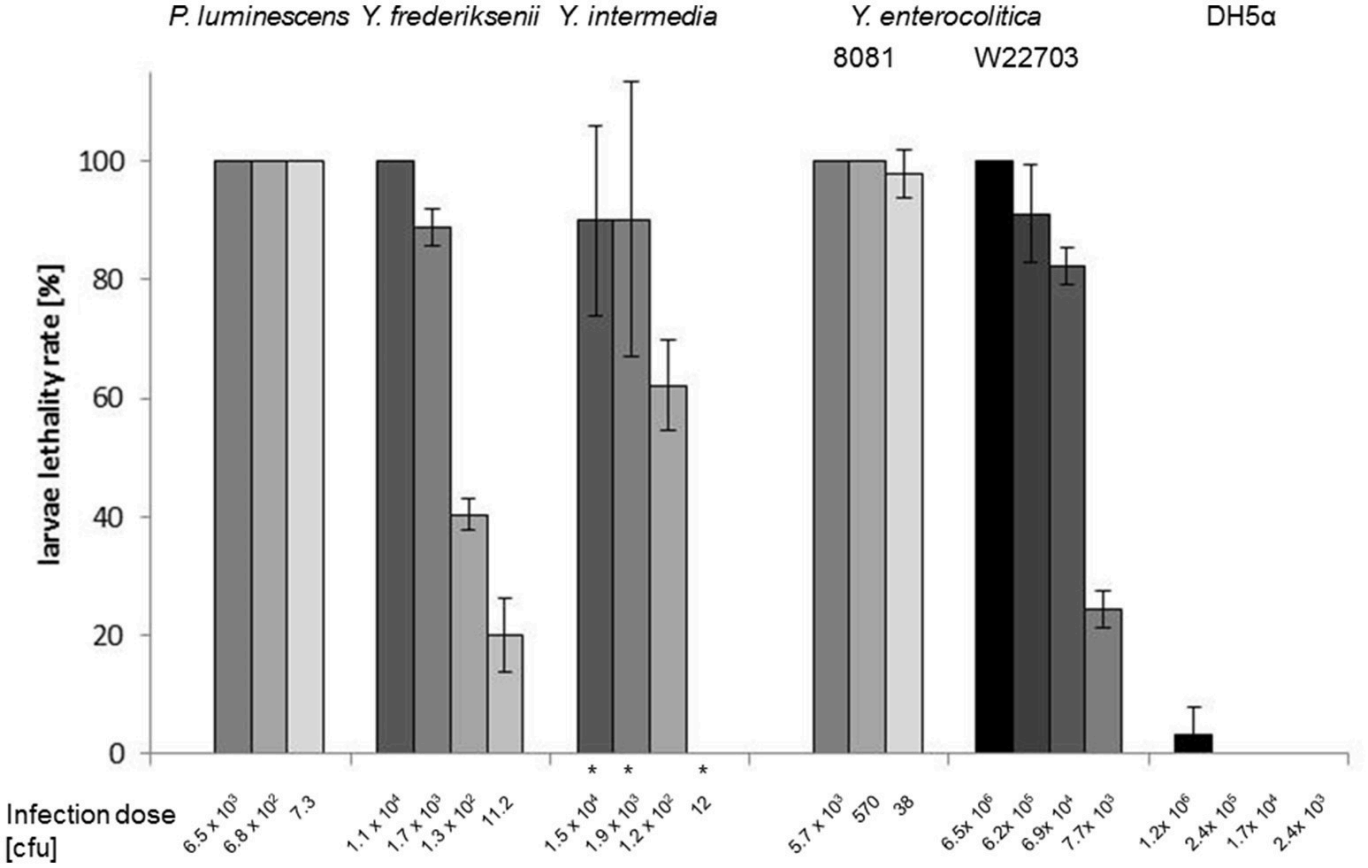
Dose-dependent toxicity of *Yersinia* strains. Survival assays were performed by infection of *G. mellonella* larvae with *P. luminescens, Y. frederiksenii, Y. intermedia, Y. enterocolitica* 8081, *Y. enterocolitica* W22703, and *E. coli* DH5α. Infected larvae were incubated at 15°C; few experiments (^*^) were performed at 20°C. The CFU used for the injection, the number of larvae, and the error bars (mean of experiments with three groups of larvae) are indicated. Groups of 15 larvae each were independently infected.

We compared the injectable insecticidal activity of *P. luminescens, Y. frederiksenii, Y. intermedia*, and *Y. enterocolitica* strain 8081 (biotype 1B, serotype O:8), which carries a so-called “high-pathogenicity island” encoding the siderophore yersiniabactin (Carniel et al., 1996). In each experiment, the lethality of the larvae decreased with lower numbers of CFU (Figure 2). Interestingly, the survival assays demonstrated that *Y. enterocolitica* 8081 is nearly as toxic as *P. luminescens* toward *G. mellonella* larvae upon intrahemocoelic infection, and is more virulent than *Y. frederiksenii* and *Y. intermedia*. Approximately 38 CFU of *Y. enterocolitica* 8081 were revealed to be sufficient to kill nearly all larvae, after an infection period of 5 days. For comparison, larvae were intrahemocoelically infected with *Y. enterocolitica* W22703 and DH5α, demonstrating the high insect-pathogenicity of *Y. frederiksenii, Y. intermedia*, and *Y. enterocolitica* strain 8081 despite the lack of TC-PAI^*Ye*^. Altogether, these data show that the lethality toward *G. mellonella* larvae is strictly dose-dependent.

### The lethality of *Yersinia* strains is temperature-dependent

**Figure 3:**
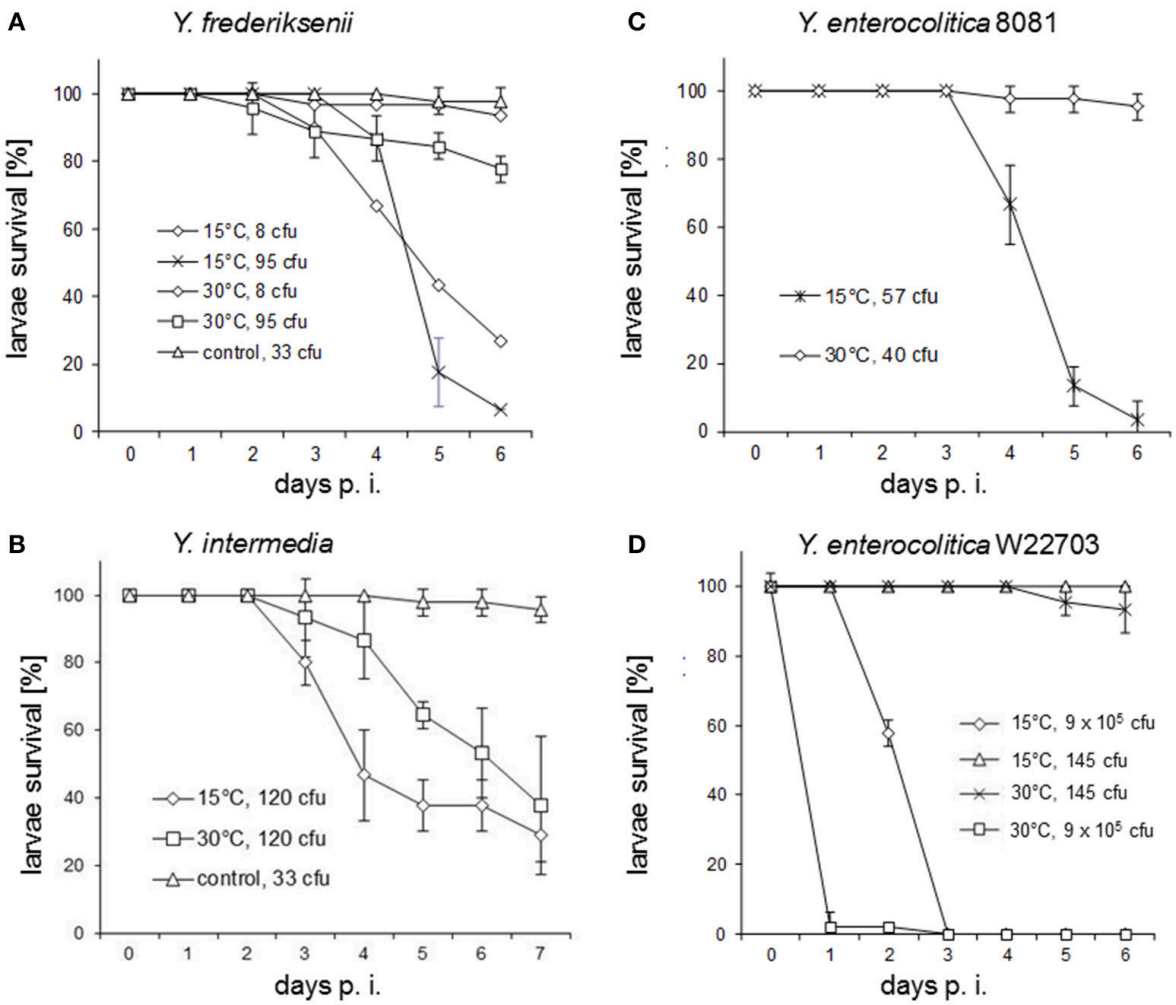
Temperature-dependent pathogenicity of *Yersinia* strains against *G. mellonella* larvae. **(A)** Larvae were each infected with eight and 95 *Y. frederiksenii* CFU, respectively, and incubated at 15°C and 30°C; the experiment at 15°C with an infection dose of eight was performed with 30 ungrouped larvae. **(B)** Infection was done with 120 *Y. intermedia* CFU and the larvae incubated at 15°C and 30°C. Infection with *E. coli* DH5α was used here as a control for all experiments. **(C)** 57 *Y*. *enterocolitica* 8081 CFU were used to infect *G. mellonella* larvae, which were incubated at 15°C; in a further assay, larvae infected with 40 CFU were incubated at 30°C; the experiment with an infection dose of 57 CFU was done with 3 × 10 larvae. **(D)** Infection assays were performed with 9 × 10^5^ or with 145 *Y. enterocolitica* W22703 CFU at 15°C and 30°C. In all experiments, three groups of 15 larvae each were independently infected with the exceptions mentioned earlier. Error bars represent the standard deviations. The larvae survival rate was plotted against day's p. i.

Low temperature-dependent toxicity of *Y. enterocolitica* W22703 toward *M. sexta* and *C. elegans*, and of representative strains of *Y. enterocolitica* phylogroups 1–5 against *G. mellonella* has been reported previously (Bresolin et al., 2006; Fuchs et al., 2008; Spanier et al., 2010; Alenizi et al., 2016). Therefore, we tested whether the injectable insecticidal activity described earlier is higher at lower temperature. *G. mellonella* larvae were infected with varying cell numbers of *Y. frederiksenii, Y. intermedia, Y. enterocolitica* 8081, and *Y. enterocolitica* W22703 and incubated at 15°C and at 30°C. However, upon infection with eight or 95 *Y. frederiksenii* CFU, the larvae showed a higher survival rate at 30°C than at 15°C (Figure 3A). A temperature-dependent pathogenicity toward *G. mellonella* was also observed for *Y. intermedia*. Although this species was found to be slightly low pathogenic at 15°C than *Y. frederiksenii*, we observed that at 30°C, 120 *Y. intermedia* CFU killed more larvae (40% survival rate) in comparison with 95 *Y. frederiksenii* CFU (80% survival rate) (Figure 3B). *Y. enterocolitica* 8081 exhibited a higher toxicity against the larvae at lower temperature as well. Only 57 CFU of this pathogen killed nearly all larvae at 15°C, but 40 CFU killed only 4% at 30°C (Figure 3C). Independent of the infection dose, *Y. enterocolitica* W22703 did not exhibit a significant temperature-dependent toxicity in this model (Figure 3D). A high infection dose of 9 × 10^5^ CFU quickly killed all larvae at 15°C and at 30°C, and a low infection dose of 145 CFU killed zero or only 7% of all larvae at these temperatures. Thus, a pronounced dose-dependent insecticidal activity was observed in these experiments with *Y. frederiksenii, Y. intermedia*, and both *Y. enterocolitica* strains.

### Phenotypes of infected larvae

**Figure 4:**
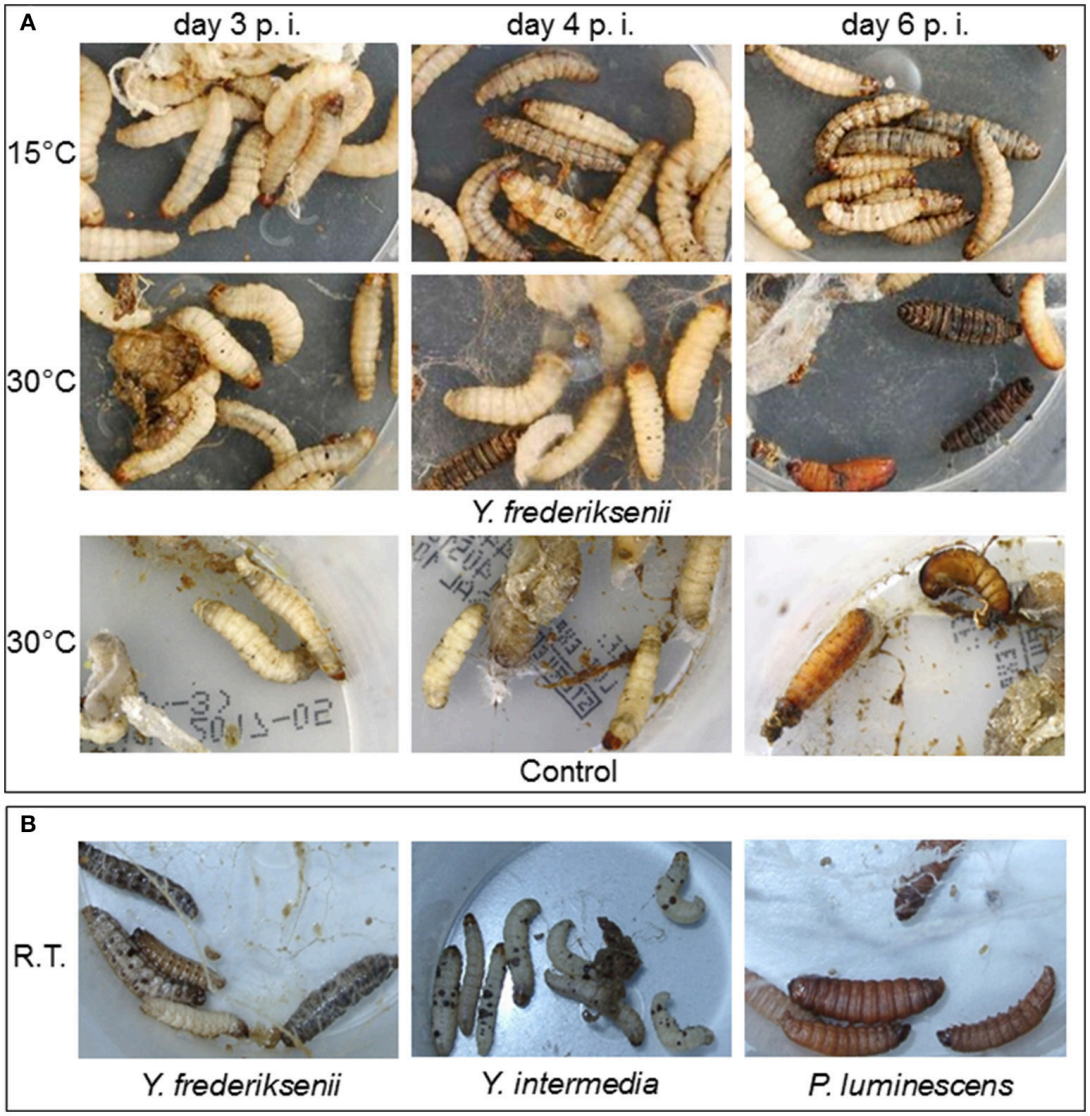
Phenotypes of *G. mellonella* larvae after infection. **(A)** Larvae were infected with 95 *Y. frederiksenii* CFU, incubated at 15°C and 30°C, and monitored until day 6 p. i.; larvae injected with 5 μl of LB medium served as control. **(B)** Infection was done with *Y. frederiksenii* (5 μl of a 10^3^ dilution of an overnight culture), with *Y. intermedia* (5 μl of a 10^4^ dilution), and with *P. luminescens* (5 μl of a 10^4^ dilution). Photographs were taken 3 days p. i. The larvae were incubated at room temperature (R.T.).

A healthy *G. mellonella* larva rapidly moves forward and back upon touch, and its exoskeleton is light colored. During the pathogenicity assays described earlier, we observed distinct phenotypes of the larvae at both 15°C and 30°C (Figure 4A). The insects infected with 95 *Y. frederiksenii* CFU were more agile at 30°C, possibly due to the lower toxicity of the pathogen at this temperature. At this temperature, injuries by combats and thus the release of hemolymph is visible from day 3 *post infectionem* (p. i.) due to the high density of insects. From day 4 to 6 p. i., the number of insects in the pupal stage as well as cocoon production increased. At 15°C, all larvae remained undamaged. However, their agility decreased from day 1 p. i. until the larvae moved only their heads or died. They also exhibited a stronger exoskeleton coloring from day 4 p. i. that strengthened until day 6 p. i. Furthermore, while non-infected larvae are sturdy, their body volume decreased upon the loss of liquid as visible in Figure 4B, left, on the larvae's surface.

Another interesting observation was the differential pigmentation of the larvae. Melanization is a defense mechanism of *G. mellonella* larvae to encapsulate pathogens, and the intensity of pigment formation correlates with the number of injected cells (Thomaz et al., 2013). Following infection with *Y. frederiksenii* and *Y. intermedia*, the larvae colored gray-brown to black before they died (Figure 4B); many larvae also exhibited a punctiform pigmentation that resembled that at the injection site. Larvae infected with *P. luminescens*, however, did not show such a pigmentation, but colored red, similar to the effect of red anthraquinones produced by *P. luminescens* following infection (Richardson et al., 1988). These observations point out to different factors that are involved in the injectable insecticidal activity of the three pathogens.

### A genome comparison approach identifies potential virulence genes present in highly and absent in weakly insecticidal strains

Although their genomes lack the *tc* genes or their homologs, *Y. intermedia* and *Y. frederiksenii* are much more toxic against *G. mellonella* larvae than *Y. enterocolitica* W22703. This finding suggests the presence of yet unknown genetic determinants that contribute to the insecticidal activity of yersinia. Therefore, we performed a genome comparison that identified 329 genes that are common for *Y. intermedia* strain ATCC 29909 and *Y. frederiksenii* strain ATCC 33641, but absent in *Y. enterocolitica* W22703 (Table S1). This set comprises a large number of genes whose (putative) products belong to categories such as lipoproteins and other membrane proteins (10 + 14), sensing, signaling, and regulation (36), metabolism (28), resistance toward toxic substances (17), transport and secretion (16 + 12), stress response (2), and iron uptake and storage (9). With respect to genetic determinants potentially involved in pathogenicity, the bioinformatics approach identified putative adhesins, toxins, hemolysins, and secretory systems (Table 2). For example, *Y. intermedia* and *Y. frederiksenii* carry a type VI secretion system (T6SS) that, among other functions, contributes to virulence (Filloux, 2013) and is present in all *Yersinia* spp. and in *P. luminescens*, but not in *Y. enterocolitica* W22703. The two species harbor an ATP-binding protein possibly involved in uptake of heme, which is absent in all other species of the *Yersinia* genus, but closely related to a protein in *Klebsiella pneumoniae*.

**Table 2:** Putative virulence factors of *Y. frederiksenii* and *Y. intermedia* absent in *Y. enterocolitica* W22703.

**Gene product**	***Y. frederiksenii^a^***	***Y. intermediam^a^***	**Closest homologs/orthologs in**	**Putative function**
ATP-binding protein	yfred0001_42840	yinte0001_30410	*Klebsiella pneumoniae*	Virulence
Enterotoxin YacT	yfred0001_650	yinte0001_42030	*Yersinia* spp. excluding *Y. pestis, Y. pseudotuberculosis, Enterococcus cloacae, P. luminescens*	*Yersinia* Ast-like cytotonic toxin
Enterotoxin	yfred0001_3400	yinte0001_16990	*Yersinia* spp. including *Y. pestis, Y. pseudotuberculosis*	Ribonuclease E
Hemolysin activator proteinlarge exoprotein	yfred0001_19600 yfred0001_19590	yinte0001_24640yinte0001_24650	*Yersinia* spp., *P. luminescens*	Heme utilization or adhesion
Thermostable hemolysin	yfred0001_34090	yinte0001_3870	*Yersinia* spp. excluding *Y. pestis* and *Y. pseudotuberculosis, Aeromonas* spp.	Cytotoxicity
Autotransporter adhesion	yfred0001_13070	yinte0001_3950	*Y. mollaretii, Serratia fonticola*	Adhesion
N-acetylglucosamine-binding protein A	yfred0001_36580	yinte0001_6590	*Yersinia* spp., *Aeromonas* spp., *Erwinia* spp., *Serratia* spp., *Pectobacterium* spp., *E. cloacae*	Adhesion
HlyD family	yfred0001_6370	yinte0001_17830	*Yersinia* spp. excluding *Y. pestis* and *Y. pseudotuberculosis, Serratia* spp.	Secretion of RTX toxin
RTX toxin and Ca^2+^-binding protein	yfred0001_38780	yinte0001_10500	*Yersinia* spp. excluding *Y. pestis* and *Y. pseudotuberculosis*, including *Y. enterocolitica* 8081	Cytotoxicity
Peroxidase-related enzyme	yfred0001_6530	yinte0001_17680	*Yersinia* spp., *Serratia* spp.	Defense
T6SS	yfred0001_31470-31660	yinte0001_22540-22350	*Yersinia* spp., *P. luminescens, Pseudomonas* spp.	Secretion of effector proteins
Twin-arginine translocation pathway signal	yfred0001_6530	yinte0001_9040	*Y. pestis* and *Y. pseudotuberculosis, Serratia* spp.	Virulence (Lavander et al., 2006)

a *Gene code was taken from the PEDANT 3 database (Walter et al., 2009)*.

### Attenuated insecticidal phenotype of *Y. frederiksenii* Δ*yacT*

**Figure 5:**
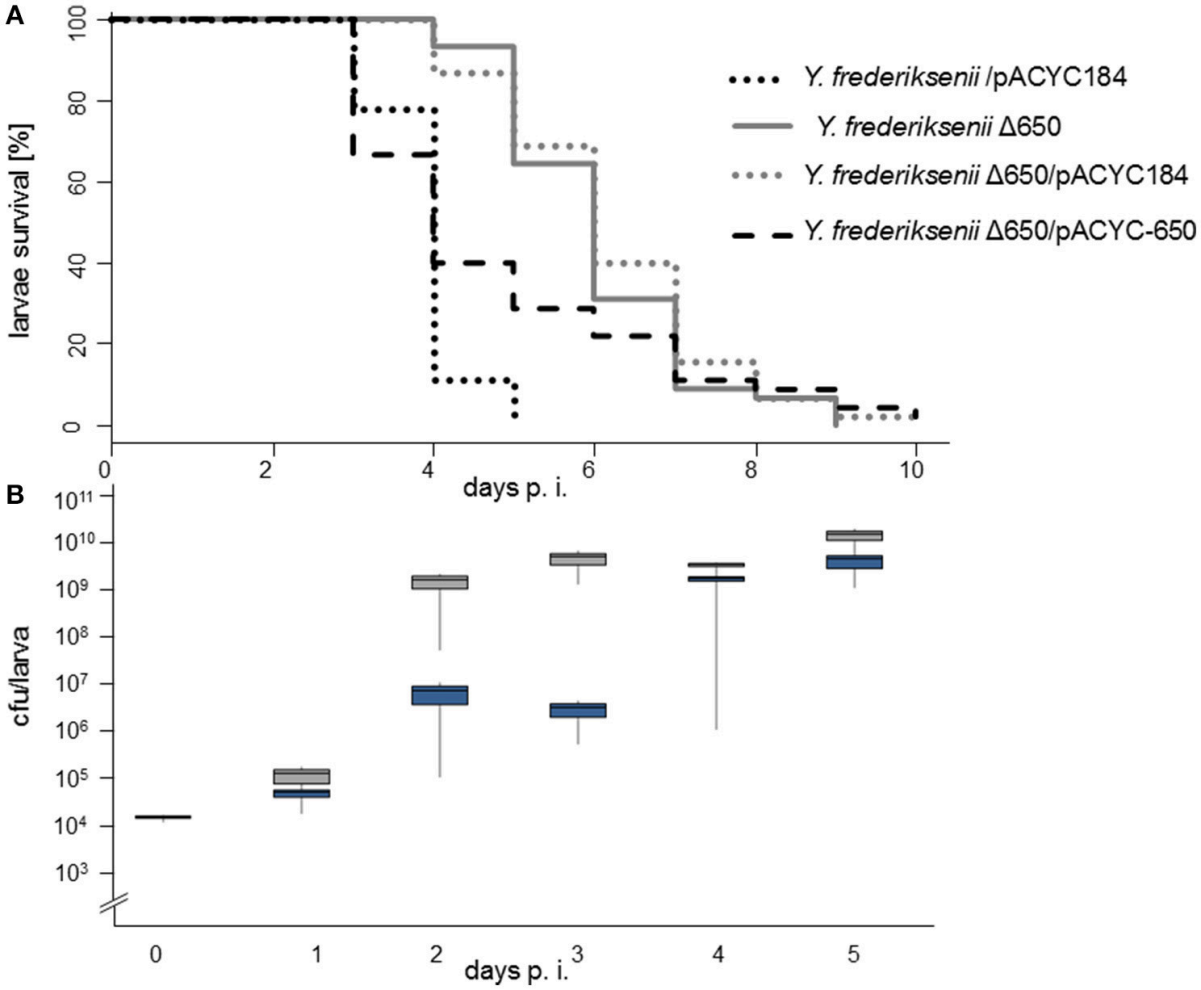
*Y. frederiksenii* Δ*yacT* exhibits attenuated virulence. **(A)** *Y. frederiksenii*/pACYC184, *Y. frederiksenii* Δ*yacT, Y. frederiksenii* Δ*yacT*/pACYC184, and *Y. frederiksenii* Δ*yacT* /pACYC-*yacT* were used to infect *G. mellonella* larvae that were incubated for 10 days at 15°C. Infection doses and TD_50_ values are indicated in Table 3. In all experiments depicted in the Kaplan–Meier plot, three independent infection experiments per strain were monitored, with groups composed of 15 larvae each. **(B)** Additionally, larvae infected in parallel were homogenized at the indicated time points and the number of viable *Y. frederiksenii* cells were enumerated. Gray boxes: *Y. frederiksenii*, black boxes: *Y. frederiksenii* Δ*yacT*. Standard deviations of three replicates are shown.

In this genome comparison approach, we identified *yacT* (accession numbers EEQ13070 and WP_004712324) encoding a protein whose amino acid sequence exhibits a significant homology (*e*-value 0.0, identity 54%; Supplementary Figure S1) to the heat-stable cytotonic enterotoxin (Ast) of *Aeromonas hydrophila*. We termed this protein, with a molecular weight of 71.46 kDa, *Yersinia* Ast-like cytotonic toxin (YacT), and the corresponding gene *yacT*. Homologs or orthologs of YacT are also encoded by *P. luminescens, P. asymbiotica*, and many *Yersinia* spp., but neither by *Y. pestis* or *Y. pseudotuberculosis*, nor by *Y. enterocolitica* strains W22703 and 8081. We generated a deletion mutant of *yacT* termed *Y. frederiksenii* Δ*yacT*, which was also complemented with pACYC-*yacT* carrying the toxin gene. On performing the *G. mellonella* infection assay at 15°C, we observed a strongly reduced virulence of *Y. frederiksenii* Δ*yacT* (time in days for 50% of the larvae to die, TD_50_ = 5.4 ± 0.22) and of *Y. frederiksenii* Δ*yacT*/pACYC184 (TD_50_ = 5.59 ± 0.01) in comparison to strain *Y. frederiksenii*/pACYC184 (TD_50_ = 3.33 ± 0.18) (Table 3, Figure 5A). When the mutant harbored gene *yacT* in trans via plasmid pACYC-*yacT*, its phenotype reverted to that of the parental strain showing a TD_50_ = 3.79 ± 0.46. These data clearly demonstrated that *yacT* is required for the high virulence of *Y. frederiksenii* toward the larvae at 15°C.

**Table 3:** Infection doses and TD_50_ values testing *yacT*.

	**CFU/ml inoculum**	**CFU per 5 μl**	TD_50_^*^(±sd)
**15**°**C**			
*Y. frederiksenii*/pACYC184	2.11 × 10^6^ ± 6.46 × 10^5^	1.05 × 10^4^	3.33 ± 0.18
*Y. frederiksenii* Δ*yacT*	2.63 × 10^6^ ± 3.05 × 10^5^	1.31 × 10^4^	5.4 ± 0.22
*Y. frederiksenii* Δ*yacT*/pACYC-*yacT*	1.94 × 10^6^ ± 3.25 × 10^5^	9.70 × 10^3^	3.79 ± 0.46
*Y. frederiksenii* Δ*yacT*/pACYC184	2.72 × 10^6^ ± 5.12 × 10^5^	1.36 × 10^4^	5.59 ± 0.01
**30**°**C**			
*Y. frederiksenii*/pACYC184	3.35 × 10^6^ ± 2.91 × 10^5^	1.68 × 10^4^	–^**^
*Y. frederiksenii* Δ*yacT*	24.11 × 10^6^ ± 7.50 × 10^5^	2.06 × 10^4^	–^**^
*Y. frederiksenii* Δ*yacT*/pACYC-*yacT*	3.90 × 10^6^ ± 3.03 × 10^5^	1.95 × 10^4^	–^**^
*Y. frederiksenii* Δ*yacT*/pACYC184	3.15 × 10^6^ ± 1.91 × 10^5^	1.58 × 10^4^	–^**^

Sd, standard deviation; ^*^, time in days for 50% of the larvae to die; ^**^, more than 50% of the larvae survived.

To understand better the role of the novel toxin during infection, the number of viable *Y. frederiksenii* cells within infected larvae incubated at 15°C was determined daily over a duration of 4 days (Figure 5B). We observed a strong growth of *Y. frederiksenii* within 4 days by more than six orders of magnitude. In comparison, a mutant *Y. frederiksenii* Δ*yacT* exhibited a retarded proliferation at day 2 p. i., followed by growth stagnation for 1 day. However, at day 4 p. i., the mutant reached approximately the same cell density as the parental strain. These data confirm that the yersiniae cell numbers increase before the larvae start to die and that YacT contributes to proliferation of *Y. frederiksenii* within the insect host.

### Effect of yacT on hemocytes

YacT was purified from *E. coli* Bl21 (DE3)/pBAD-HisA(tet)-650. Six microliter of a toxin solution with a concentration of 1.4 μg/μl or of phosphate-buffered saline (PBS) as control were injected into 20 *G. mellonella* larvae. The larvae of the toxin group showed paralysis of the half retral abdomen immediately after injection. In addition, some caterpillars of this group displayed a constriction of the head-thorax area and did not react to touching. In comparison, the control group showed none of these symptoms. One day p.i., animals of both groups that were kept at 30°C maintained vigor and formed fine webs. After web removal, 2 or 3 days p. i., the caterpillars of the toxin-treated group showed punctate- to strokelike black discolorations at the dorsal–abdominal areas that we did not observe in the control group (Supplementary Figure S2).

**Figure 6:**
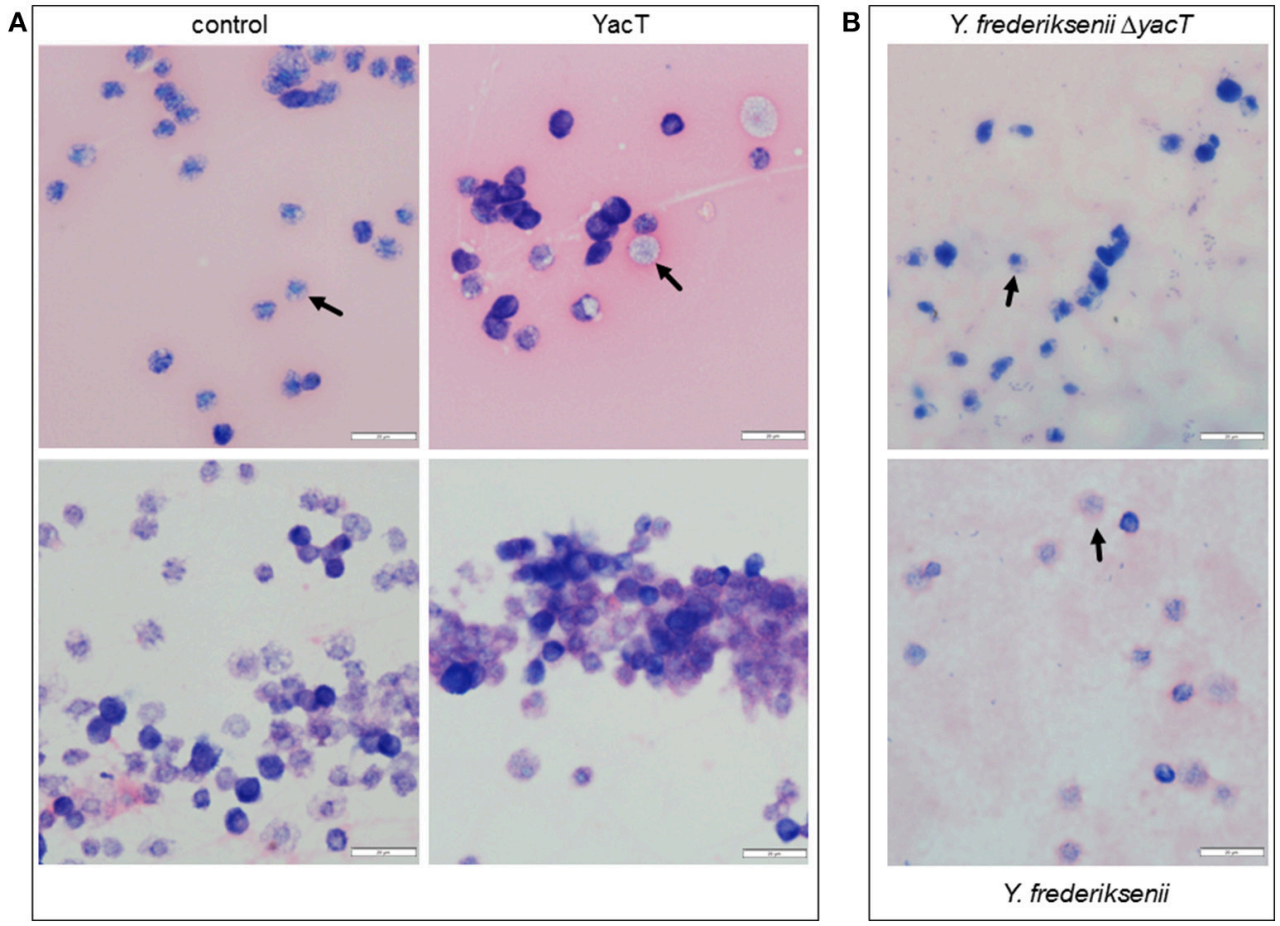
Effect of purified YacT of *Y. frederiksenii* on hemocytes. **(A)** Six Microliter of purified YacT or of PBS (control) were injected. After 1 day, hemolymph preparations of *G. mellonella* larvae were fixed with methanol (upper row) or with aceton (lower row) and then stained by Giemsa solution. The hemocytes derived from YacT-treated larvae began to form some aggregates in comparison with controls (lower row). **(B)** Hemolymph preparations of larvae 1 day after oral infection with 6 μl of an overnight culture of *Y. frederiksenii* and *Y. frederiksenii* Δ*yacT*. Arrows point to changes of hemocyte morphology and chromatin density, the latter one as visible by the weaker nuclear staining. Photos of representative preparations are shown; the scale is indicated. Microscope Olympus BX53 was used with 600 × magnification.

To study the effect of YacT on hemocytes after 1 day, the hemolymph of larvae was prepared from the aorta and streaked out on microscope slides for staining. Injection of PBS (10 mM phosphate buffer, pH 7.4; 2.7 mM KCl; 137 mM NaCl) served as a control. Upon microscopic analysis, we observed repeatedly several distinct cell phenotypes: hemocytes from animals treated with the toxin showed a round-shaped morphology and they began to form aggregates in comparison to the controls and several cells also enlarged and showed a reduction of chromatin, possibly indicating the beginning of early stages of the cell death (Figure 6A).

As a complementary experiment, *G. mellonella* larvae were infected orally with *Y. frederiksenii* and its *yacT* deletion mutant. Again, the morphology and chromatin density of hemocytes were modified in the presence of *Y. frederiksenii*, but not of *Y. frederiksenii* Δ*yacT* (Figure 6B). In both cases, yersinial cells were visible in the preparations, indicating that penetrating the gut epithelial barrier occurs independent of YacT.

## Discussion

Members of the genus *Yersinia* are fascinating organisms, as they are able to adapt to the environmental life cycle stage as well as to mammals (Fuchs et al., 2011). During a transition, they encounter a broad spectrum of hostile conditions, and a major clue to overcome these challenges is the temperature-dependent production of host-specific virulence factors. Therefore, the interaction of yersiniae with invertebrates may have been a precursor to human pathogenicity during evolution (Waterfield et al., 2004). In this study, we tested the entomopathogenic potential of a set of *Yersinia* spp. toward larvae of *G*. *mellonella*. The larvae are considered to be a natural host of yersiniae and other pathogens and, therefore, serve as an indicator of yersinial virulence activities against insects. We identified *Y. enterocolitica* 8081, a representative of the highly pathogenic biovar 1B group, to be the most virulent *Yersinia* strain tested so far against *G*. *mellonella* larvae, resembling the high insecticidal activity of *P. luminescens*. Data on strain 8081 as the least pathogenic strain among several *Y. enterocolitica* strains tested against *G. Mellonella* are not in contradiction with our findings, because Alenizi et al. performed the infection experiments at 25°C and missed the high toxicity at the environmental temperature of 15°C (Alenizi et al., 2016). *Y. enterocolitica* strain 5303, which belongs to the biovar 1A group and is considered to be apathogenic toward mammals, showed an even higher toxicity toward the *Galleria* larvae, since only ten CFU were sufficient to kill 50% of the larvae within 5 days (Alenizi et al., 2016). Interestingly, also *Y. intermedia* and *Y. frederiksenii* are more virulent to *Galleria* larvae than other *Yersinia* spp., including *Y. mollaretii, Y. bercovieri, Y. ruckeri, Y. aldovae*, and *Y. kristensenii*, as tested recently (Fuchs et al., 2008). *Y. frederiksenii* and *Y. intermedia* occupy related ecological niches and exhibit very similar phenotypes (Martin et al., 2009). *Y. intermedia*, which is isolated mainly from the environment, animals, food, and (rarely) human beings, received its name due to its genetic and phenotypic properties that are an intermediate between those of *Y. pseudotuberculosis* and *Y. enterocolitica* (Martin et al., 2009). *Y. intermedia* also shares several O antigens with *Y. enterocolitica* (Wauters et al., 1972), of which O:4 and O:17 are probably the prevailing serotypes (Ursing et al., 1980). *Y. frederiksenii* was differentiated from *Y. enterocolitica* in 1980 (Ursing et al., 1980). The high insecticidal potential might point out to yet overlooked natural habitats of these strains.

Temperature is an important signal in the regulation of yersinia virulence factors of that are predominantly produced at 37°C and repressed at temperatures lower than body temperature, or vice versa, as exemplified by the insecticidal *tc* genes in *Y. enterocolitica* W22703. Temperature-dependent mortality of *G. mellonella* upon oral infection, but not upon intrahemocoelic injection, was observed recently for *Y. entomophaga* (Hurst et al., 2015). Therefore, it is not a surprising outcome of this study that the toxicity of *Y. frederiksenii, Y. intermedia*, and *Y. enterocolitica* 8081 increases with lower temperature, thus pointing out to a relevant ecological niche of these strains. Irrespective of the fact that *G. mellonella* has been chosen here as an infection model rather than as a natural host for yersinial infection, the two temperatures applied here correspond to the lifestyle of *G. mellonella* larvae that grow best between 29°C and 35°C, and also develop at 15°C, but not at 10°C or less.

The pronounced contrast between the insecticidal potential of *Y. frederiksenii, Y. intermedia*, and *Y. enterocolitica* 8081, on the one hand, and the TC-PAI^*Ye*^-harboring *Y. enterocolitica* W22703, on the other hand, at least with respect to the *G.mellonella* model used here, prompted us to perform a genome comparison. This approach aimed to identify the determinants that confer the high insecticidal activity of these strains. Table 2, which probably still lacks several factors involved in infection, points out to a broad spectrum of yersinial factors whose role in pathogenicity as well as their host specificity remains to be investigated. One of them is YacT that is highly homologous to the *ast*-encoded heat-stable, cytotonic enterotoxin of *A. hydrophila* (Chopra et al., 1994) that was associated with gastroenteritis and non-bloody diarrhea in children and shown to contribute to the fluid secretory response in a murine model (Sha et al., 2002). Cell lysates of *E. coli* cells carrying *ast* elongated Chinese hamster ovary cells, which is a typical response to enterotoxins (Chopra et al., 1994). Besides *Yersinia* strains, YacT orthologs were identified also in *P. luminescens* ssp. *laumondii* (*e*-value = 10^−177^) and in the human pathogen, *P. asymbiotica* (*e*-value = 10^−179^), demonstrating that this factor is not unique to *A. hydrophila* as assumed previously (Sha et al., 2002). The prevalence of the Ast and YacT homologs confirms the strong functional relatedness between *Photorhabdus* spp. and *Yersinia* spp. with respect to their invertebrate and vertebrate association (Heermann and Fuchs, 2008). It is important to note that YacT is distinct from the heat-stable enterotoxin Yst of yersiniae, for which a homolog is missing in *Y. frederiksenii* ATCC 33641 and *Y. intermedia* (Singh and Virdi, 2004). Our data demonstrate that YacT is required for full pathogenicity toward *G. mellonella*. Moreover, the finding that YacT injection affects the morphology of hemocytes suggests that the immune response of *G. mellonella* controls better the proliferation of *Y. frederiksenii* Δ*yacT* during the first 3 days p. i. as compared with that of *Y. frederiksenii*. The list of determinants in Table 3 and the variation of *Yersinia* spp. in pathogenesis toward *Galleria* larvae suggest that the yersinial toxicity toward insects upon intrahemocoelic infection is a multifactorial process due to the presence of several cytotoxic determinants. In the light of this assumption, the virulence attenuation upon deletion of *yacT* in *Y. frederiksenii* is remarkably high. Therefore, YacT is a candidate to explain the high toxicity of *Y. frederiksenii* against *G. mellonella*.

## Conclusion

A major implication of this study is that the yersinial toxicity toward insects not only depends on the TC, but also on a broader set of insecticidal toxins than known so far. We identified a novel yersinial entomopathogenic factor, whose activity might be associated with the hemocoel rather than with the insect gut as indicated by the distinct oral and intrahemocoelic toxicity of *Y. intermedia* and *Y. frederiksenii*. The findings of this study and other studies suggest that yersiniae strains, regardless of being human pathogens or not, acquire a substantial selection advantage by entering invertebrates. By overcoming infection barriers such as the gut epithelium or the innate immune response of insect larvae or nematodes, they might bioconvert their host, thus getting easy access to energy- and nitrogen- rich nutrients. The resulting proliferation increases the chance of *Yersinia* strains to be transmitted to other hosts including mammals.

## Materials and methods

### Bacterial strains and growth conditions

The strains used in this study were *Y. intermedia* (Collection Institut Pasteur [CIP] 80.28; ATCC 29909), *Y. frederiksenii* (CIP 80.29; ATCC 33641), *Y. enterocolitica* 8081 (Virginia Miller, St. Louis, USA), *Y. enterocolitica* W22703 (Cornelis and Colson, 1975), and *P. luminescens* ssp. *laumondii* strain TT01 BX470251 (Fischer-Le Saux et al., 1999). All cultures were grown in lysogeny broth (LB) (10 g l^−1^ tryptone, 5 g l^−1^ yeast extract, and 5 g l^−1^ NaCl) or on lysogeny broth (LB) agar (LB broth supplemented with 1.5 % w/v agar). *Escherichia coli* were grown at 37°C and *P. luminescens* and *Yersinia* strains at 30°C. If appropriate, the media were supplemented with the following antibiotics: 50 μg ml^−1^ streptomycin, 12 μg ml^−1^ tetracycline, 50 μg ml^−1^ kanamycin, 20 μg ml^−1^ chloramphenicol, and 20 μg ml^−1^ nalidixic acid.

### General molecular techniques

The DNA manipulation was performed according to standard procedures (Sambrook and Russell, 2001). To isolate the chromosomal DNA, 1.5 ml of a bacterial culture was centrifuged, and the sediment was re-suspended in 400 μl of lysis buffer (100 mM Tris pH 8.0, 5 mM EDTA, 200 mM NaCl). After incubation for 15 min on ice, 10 μl of 10% SDS and 5 μl of proteinase K (10 mg/ml) were added, and the sample was incubated overnight at 55°C. The chromosomal DNA was precipitated with 500 μl of isopropanol, washed in ethanol, dried, and dissolved in 500 μl of TE buffer (10 mM Tris-HCl, 1 mM Na_2_EDTA, pH 7.4) containing 1 μl of RNase (10 mg/ml). Polymerase chain reactions (PCR) were carried out with Taq polymerase (Fermentas, Vilnius, and Lithunia) and the following programme: one cycle at 95°C for 2 min; 30 cycles at 95°C for 10 s, at the appropriate annealing temperature for 30 s, at 72°C for 45 s to 180 s depending on the expected fragment length; one cycle at 72°C for 10 min. Four Microliter of chromosomal DNA (100 ng ml^−1^) was used as a template for PCR amplification, and the GeneRuler DNA mix (Fermentas) served as a DNA ladder.

### Genome comparison

The sequences of genome used for the comparison were that of *Y. enterocolitica* 8081 (accession numbers AM286415 for the chromosome and AM286416 for the plasmid), *Y. intermedia* (genome draft: GCA_000168035.1), and *Y. frederiksenii* (genome draft: GCA_000754805.1). Homology searches of predicted proteins were performed by basic local alignment search tool analysis (Altschul et al., 1997). The PEDANT software system [http://pedant.gsf.de; (Walter et al., 2009)] was used for automatic genome sequence analysis and annotation (Frishman et al., 2001). Genomes were recorded and homology searches of predicted proteins were performed by SIMAP (Arnold et al., 2014). The genome comparisons were calculated by using a custom Perl script, which formatted bidirectional-best sequence hits between all predicted proteins (*E* ≤ 0.0001).

### Insecticidal bioassays

*M. sexta* were reared as described (Schachtner et al., 2004). For oral bioassays, bacteria were grown at 15°C (*Yersinia* strains) or 37°C (DH5α) until stationary phase. About 50 μl of a culture was applied to 4 mm^3^ disks of an agar-based artificial diet (David and Gardiner, 1965). The liquid was allowed to soak into the agar block, which was dried under a laminar flow. First-instar *M. sexta* neonate larvae were placed on the disk and incubated at 22°C. The application of bacterial culture aliquots was repeated after 3 days, and the larvae mortality was recorded after 5 days.

Larvae of *G. mellonella* were obtained from the Zoo-Fachmarkt (München, Germany) and stored for less than 1 week at room temperature. Bacterial strains were grown to stationary phase (optical density at 600 nm [OD_600_] ~1–5 × 10^9^ cfu/ml) at temperatures between 15°C and 30°C (*Yersinia* spp.), at 30°C (*P. luminescens*), or at 37°C (DH5α), and 10-fold serial diluted. Larvae of 2–3 cm length and of 110–130 mg weight were used. A 5 μl of the bacterial culture or an appropriate dilution thereof were orally applied or injected by a sterilized microsyringe (Hamilton 1702 RN, 25 μl) into the hemocoel through the last left proleg. The aperture reseals after the removal of the syringe, thus preventing the loss of inoculum (Kavanagh and Reeves, 2004).

Infection doses were determined by plating serial dilutions of the cultures used for injection. Control assays had demonstrated that neither the medium nor the wounding by the syringe contributes to the mortality rate of the insects (Fuchs et al., 2008). Infected larvae were incubated for at least 5 days in the dark at the temperature indicated and the number of killed and alive larvae were enumerated each day. Larvae were considered dead if they failed to respond to touch. The TD_50_ was calculated using the dose-response curve (drc) package of the R software. To recover bacteria from the larvae, the larvae were surface sterilized with 70% ethanol, washed in H_2_O, and cut into small pieces. The homogenous mass was suspended into 1 ml LB, rigorously shaken for 5 min with a vortex, and centrifuged at 1,000 rpm for 2 min. Serial dilutions were plated on agar plates with LB or with *Yersinia* selective medium (Schiemann CIN medium, Oxoid, Wesel, Germany).

### Deletion mutants and complementing plasmid

In-frame deletion of *yacT* from *Y. frederiksenii* was performed by the one-step method based on the phage λ Red recombinase (Datsenko and Wanner, 2000). In short, PCR products comprising the kanamycin resistance cassette of plasmid pKD4, including the flanking FRT sites, were generated using pairs of 70-nucleotide-long primers that included 20 nucleotides priming sequences for pKD4 as template DNA. Homology extensions of 50 bp overlapped 18 nucleotides of the 5′-end and 36 nucleotides of the 3′-end of the target gene (Link et al., 1997). About 500–1,000 ng of fragment DNA were transferred into *Y. frederiksenii* cells harboring plasmid pKD119. Allelic replacement of the target gene by the kanamycin resistance cassette was controlled by PCR, and nonpolar deletion mutants were obtained via transformation of pCP20. The deletion was confirmed by PCR and sequencing.

Gene *yacT* including 220 bp upstream and 100 bp downstream of the coding sequence was amplified with the oligonucleotides 5′-CGATGAATTCAGTGACCGTCTGTGGGTCTG-3′ and 5′-CGGCCATGGGGGGGCAGCATCGTGGATTC-3′ and ligated into the chloramphenicol resistance cassette of plasmid pACYC184 via *Nco*I und *EcoR*I, resulting in pACYC-*yacT*. The recombinant plasmid was validated by PCR and sequencing.

### Overproduction and purification of yacT

Gene *yacT* of *Y. frederiksenii* was cloned into plasmid pBAD-HisA(tet) (Starke et al., 2013) *via Sac*I and *Pst*I using the oligonucleotides 5′-CGATGAGCTCATGCAGAAAATCATACCGAG-3′ and 5′-AACTGCAGTTATTGGGTGCTAGCCACAG-3′. An overnight culture of *E. coli* Bl21 (DE3)/pBAD-HisA(tet)-650 was diluted 1:100 into 800 ml of LB medium supplemented with 12 μg/ml tetracycline and incubated at 37°C with rotation at 180 rpm. At an OD_600_ of 0.6, protein production was induced by adding 0.2% of arabinose. After incubation for an additional 4 h at 37°C and 180 rpm, the cells were harvested by centrifugation at 4°C and 7,500 rpm for 20 min. The pellets were each re-suspended in 5 ml of native lysis buffer (50 mM NaH_2_PO_4_, 300 mM NaCl, and 10 mM imidazole at pH 8.0) in the presence of 1 mM protease inhibitor Pefabloc SC (Sigma-Aldrich, Taufkirchen, Germany) and lysed by 4 passages through a French press (SLM Aminca Instruments, Rochester, NY, USA) at 900 psi; residual cell debris was removed thrice by centrifugation at 4°C and 9,000 rpm for 15 min. Following the filtration, YacT was isolated using the Ni-NTA Fast Start Kit (Qiagen, Hilden, Germany) according to the manufacturer's instructions. For imidazole removal, proteins were dialyzed against 50 mM phosphate buffer plus 0.5 mM MgSO_4_, 0.5 mM ZnSO_4_, and 0.5 mM CaCl_2_ and protein extracts were concentrated down to 1 ml with Amicon ultracentrifugal filter units (Millipore). The protein concentration was determined using Roti-Quant solution (Carl, Roth GmbH, Karlsruhe, Germany) according to the Bradford method (Bradford, 1976). The purity of the eluted fractions was analyzed by the separation on a 12.5% sodium dodecyl sulfate (SDS)-PAA gel (Supplementary Figure S3).

## Author contributions

KS, P-AS, and CM performed infection assays and analyzed the results, AF constructed the recombinant strains, TR was responsible for the genome comparison, and TF analyzed the data, conceived the study, and wrote the manuscript. All authors drafted and revised the work and approved of the final version.

### Conflict of interest statement

The authors declare that the research was conducted in the absence of any commercial or financial relationships that could be construed as a potential conflict of interest.
