# Supplementary material for: Insecticidal Toxicity of Yersinia frederiksenii Involves the Novel Enterotoxin YacT
Source: Front Cell Infect Microbiol. 2018 Nov 14;8:392. doi: 10.3389/fcimb.2018.00392 (PMC6246891; doi:10.3389/fcimb.2018.00392)
Supplement: Supplementary file 1 [file Data_Sheet_1.pdf]

Table S1. Products of genes present in *Y. frederiksenii* and *Y. intermedia*, but absent in *Y. enterocolitica* 22703.

Gene product	Comment	Category
Ferrichrysobactin receptor		Iron metabolism
AMP-dependent synthetase and ligase	Degradation of heme to bilirubin	Iron metabolism
Hemin transport protein hmuS	Hemin uptake	Iron metabolism
4Fe-4S ferredoxin, iron-sulfur binding	Intracellular sensing	Iron metabolism
HmsH protein	Hemin storage	Iron metabolism
Nodulin 21-like protein	Nodulin/iron and manganese transporter domain	Iron metabolism
Dipeptide ABC transporter, ATP-binding protein	Putative heme uptake	Iron metabolism
Dipeptide ABC transporter, ATP-binding protein	Putative heme uptake	Iron metabolism
Iron-binding protein IscA	Iron-sulfur cluster assembly scaffold	Iron metabolism
Lipopolysaccharide biosynthesis protein RffA		Lipoprotein
PutativeLipoprotein		Lipoprotein
Putative Lipoprotein		Lipoprotein
Putative Lipoprotein		Lipoprotein
Lipid A biosynthesis lauroyl acyltransferase		Lipoprotein
Polymerase Wzy	O-antigen biosynthesis protein	Lipoprotein
Uncharacterized lipoprotein YfhM	Cell membrane; lipid-anchor	Lipoprotein
Outer membrane lipoprotein carrier protein LolA	Sorting of lipoproteins	Lipoprotein
Putative lipoprotein		Lipoprotein
Glycosyl transferase family 2	LPS biosynthesis	Lipoprotein
Putative inner mebrane protein		Membrane
Conserved integral membrane protein		Membrane
Porin B		Membrane
Putative inner membrane protein		Membrane
Putative outer membrane protein		Membrane
Outer membrane pore protein E		Membrane
Outer membrane protein S1		Membrane
Inner membrane protein YebS	Involved in phosphotransfer	Membrane
Putative membrane protein Ycjf		Membrane
OmpA domain protein		Membrane
Putative transmembrane protein		Membrane
Inner membrane protein YcdZ		Membrane
Membrane receptor protein		Membrane
Inner membrane protein yecN	Three predicted transmembrane domains	Membrane
Ethanolamine ammonia-lyase heavy chain	Degradation of amines	Metabolism
Ethanolamin permease		Metabolism
Ethanolamine ammonia-lyase light chain		Metabolism

Tetrathionate reductase subunit A		Metabolism
L-fucose isomerase		Metabolism
L-fucose operon activator		Metabolism
Fucose isomerase domain protein		Metabolism
L-fucose-proton symporter		Metabolism
L-fucose mutarotase		Metabolism
L-rhamnose isomerase		Metabolism
L-rhamnose mutarotase		Metabolism
L-rhamnose-proton symporter		Metabolism
Cobalamin biosynthesis protein cbiB		Metabolism
N-acetylglucosamine-6-phosphate deacetylase		Metabolism
N-acetylmannosamine-6-phosphate 2-epimerase		Metabolism
Vitamin B12 import ATP-binding protein BtuD		Metabolism
Vitamin B12-binding protein		Metabolism
Ammonia channel		Metabolism
DsdX permease	Gluconate transport	Metabolism
α-ketoglutarate permease		Metabolism
Xylose isomerase domain protein TIM barrel		Metabolism
Formate transporter 1		Metabolism
DeoX-like	Deoxyribose mutarotase	Metabolism
Cellulase synthase operon protein		Metabolism
Thiamine biosynthesis lipoprotein ApbE		Metabolism
Ornithine carbamoyltransferase		Metabolism
Inducible histidine transporter		Metabolism
Histidine utilization repressor		Metabolism
β-lactamase domain protein	Beta-lactamase	Resistance
Antibiotic biosynthesis monooxygenase	Biosynthesis of several antibiotics	Resistance
AbgT family protein	Putative drug efflux pump	Resistance
Acriflavine resistance protein B		Resistance
Uncharacterized inner membrane transporter YhbE	DMT family, putative drug permease	Resistance
Innermembrane transport protein YdhC	Putative drug resistance transporter	Resistance
Uncharacterized GST-like protein YghU	Putative detoxification of xenobiotics	Resistance
Arsenical pump membrane protein		Resistance
Integral membrane protein	DMT family, putative drug efflux pump	Resistance
Lactoylglutathione lyase	Detoxification of methylglyoxal	Resistance
Microcin-24	Bacteriocin	Resistance
Heavy metal efflux pump, CzcA family		Resistance
Glyoxalase/bleomycin resistance		Resistance
Cation/multidrug efflux pump	-	Resistance
Uncharacterized GST-like protein YghU	Putative detoxification of xenobiotics	Resistance
Multidrug resistance protein MdtG	Resistance to bile salt and fosfomycin	Resistance
Arsenate reductase		Resistance

PTS permease protein	Phosphotransferase system	Secretion
YeeE/YedE	Secretory pathway	Secretion
Twin-arginine translocation pathway signal	Transport of folded protein	Secretion
Efflux transporter, RND family, MFP subunit		Secretion
Tricarboxylic transport		Secretion
Efflux transporter, RND family		Secretion
Tricarboxylic transport		Secretion
Tricarboxylic transport		Secretion
YeeE/YedE family protein	Secretory pathway	Secretion
Arginine-binding periplasmic protein 1	ABC-transporter	Secretion
Magnesium transporter		Secretion
Outer membrane efflux protein	Similar to heavy metal RND efflux OMP	Secretion
Two-component sensor kinase		Sensing, signalling and regulation (SSR)
Carbonic anhydrase	CO2 sensing, putative role in pH regulation and virulence induction	SSR
Transcriptional regulatory protein BaeR		SSR
Transcriptional regulatory protein		SSR
HTH-type transcriptional regulator DsdC		SSR
Starvation-sensing protein rspA	Putative HSL degradation	SSR
3-oxoacyl-[acyl-carrier-protein] reductase		SSR
AraC-family transcriptional regulator	Putative stress response	SSR
Sensor kinase dpiB		SSR
Putative transcriptional regulator		SSR
Transcriptional regulatory protein TctD		SSR
3-oxoacyl-[ACP] synthase		SSR
Transcriptional regulatory protein		SSR
Transcriptional regulator	*B. thuringiensis* regulator MarR	SSR
3-oxoacyl-[acyl-carrier-protein] reductase	Involved in covalent fatty acid acylation of RTX-toxins	SSR
Acetoacetate metabolism regulatory protein AtoC		SSR
HTH-type transcriptional activator RhaR		SSR
Response regulator		SSR
Transcriptional regulator, LysR family		SSR
PAS/PAC domain		SSR
LysR-family regulatory protein		SSR
Signal transduction histidine-protein kinase AtoS		SSR
HTH-type transcriptional activator RhaS		SSR
Diguanylate cyclase with PAS/PAC sensor		SSR
Transcriptional regulatory protein		SSR
AraC-type DNA-binding domain-containing protein		SSR

Transcriptional regulator, LysR family		SSR
Predicted transcriptional regulator	DeoR family, similar to *Legionella* regulator	SSR
Predicted transcriptional regulator	MerR family regulator, putative response to oxidative stress, heavy metals	SSR
Transcriptional regulator		SSR
Sensor protein RstB		SSR
Two-component system response regulator		SSR
Transcriptional regulator		SSR
Putative kinase inhibitor	Phospholipid-binding protein	SSR
HTH-type transcriptional regulator TreR		SSR
Predicted transcriptional regulator		SSR
Universal stress protein F		Stress response
Heat shock protein 15		Stress response
Member of the Nips/Nap superfamily	Putative role in vesicular transport	Transport
Uncharacterised symporter YihO	GPH-cation symporter family	Transport
Extracellular solute-binding protein family 5	ABC-transporter	Transport
Lysine-specific permease		Transport
MFS-family transporter	Secondary active transport	Transport
D-galactonate transporter		Transport
Arabinose import ATP-binding protein araG 2	ABC-transporter	Transport
Sugar ABC transporter, periplasmic protein		Transport
Sugar ABC transporter		Transport
ABC transporter related		Transport
Arginine/ornithine antiporter	ABC transporter	Transport
Ribose ABC transporter		Transport
Short-chain fatty acids transporter		Transport
Inner-membrane translocator		Transport
PTS system, glucose-like		Transport
