# Supplementary material for: Insecticidal Toxicity of Yersinia frederiksenii Involves the Novel Enterotoxin YacT
Source: Front Cell Infect Microbiol. 2018 Nov 14;8:392. doi: 10.3389/fcimb.2018.00392 (PMC6246891; doi:10.3389/fcimb.2018.00392)

Supplementary Figure S1: Homology between YacT and Ast

```
ACCESSION #:    WP_101615653 Aeromonas hydrophila
                EEQ13070 Yersinia frederiksenii ATCC 33641

E value: 0.0; Query cover: 90%; Identity: 54%

Data-Reference: https://blast.ncbi.nlm.nih.gov/Blast.cgi


A. hydrophila       1 MHARTAMTLLLMLTP-FAQAETLSLEGDQYRIKVETDAGKFRELTFTNTANGDLIRSDN-
Y. frederiksenii    1 MQKIIPSSLLLLLMSHGAFAAEYLLNNDKLSLSFD------------D-KNSAVVVKDKL
consensus           1 *      .***.*  . * *    *  *   .  ............. . *  ..  * .

A. hydrophila      59 ---------LFDIKGTDDRLTPSADFKVTAFTKSDDRIEMALENADFVVKSAIRLGRDKR
Y. frederiksenii   48 SDHKLAPVELFFLTLPNEDVIHAADFNIKNVTQKDDAIHIDYEHKDFDVTVVLNVLKG-K
consensus          61 .........** .    . .   *** .   *  ** * .  *  ** *   . . . ..

A. hydrophila     110 YASIDFALDTLKNGKKINGFSMLPF--HSQAPFVYGAINSSPIVSDSFFITPQNPLVNTR
Y. frederiksenii  107 YASIDYTIKAKGKARDVAKITFFPTKGQSQAPYVDGAINSSPIIADSFFILPEKPIVNTY
consensus         121 *****. .     .. .   .  * .. ****.* ********. ***** *  *.***

A. hydrophila     168 AYEGGVSQLIPLKLPLAEGKPLSYRTYVGTFGE-GQLRRDFNRFLNEARDRPYAPYLHYN
Y. frederiksenii  167 AYETTTNLNVELKTPIETDAPVTFTTYFGTFEENNQLRRSFNQFINAMRPRPYQQYLHYN
consensus         181 ***      . ** *.    *... ** *** *. **** ** *.*  * ***  *****

A. hydrophila     227 SWLDIGFFNPYTEAEALKRIDQFGEALISRRGVPMNGFLFDDGWDDRLGNWGFSKDFPNG
Y. frederiksenii  227 SWMDIGFFTTYTEQEVLDRMDTYADELMKKRGVQLDGFLLDDGWDDRTGKWLFGPAFSKG
consensus         241 **.*****  *** * * *.* ... *. .*** . *** ******* * * *   *  *

A. hydrophila     287 FSKLKRAAERYHAQLGIWLSPWGGYNKPRDERVSHAAEFGYELSDGKFALSGPVYYQNFH
Y. frederiksenii  287 FSVVKEKADSLNTSVGLWLSPWGGYNKPRDIRVSHAKENGFETVDGKFALSGPNYFRNFN
consensus         301 ** .*  *.     .*.************* ***** * *.*  ********* *. **

A. hydrophila     347 QKVLSLIKDQGVSHFKFDGTGNADKLIEGSRFTSDFDAAIHLIADARAANPKVFINLTTG
Y. frederiksenii  347 EQIIKLIKNEHITSFKLDGMGNANSHIKGSQFASDFDASIELIKNMREANKDLFINLTTG
consensus         361   .. ***   .. ** ** ***   * ** * ***** * **   * **  .*******

A. hydrophila     407 TTASPAWLFFADSIWRGGDDINFYGPGSRVQQWITYRDAETYRSIVKNGPLFPLNSLMLH
Y. frederiksenii  407 TNASPSWLFYADSIWRQGDDINVFGKGSPAQQWMTYRDAETYRSIVRKGPLFPLNSLMYH
consensus         421 * *** ***.****** ***** .* **  ***.************. ********** *

A. hydrophila     467 GLVYAKQAKH-LD-RQSPQDFADQAWSYFATGTQLQELYLTPELLSGQNWDLLAKAALWS
Y. frederiksenii  467 GIVSAENAYFGLEKVQTDPDFADQVWSYFATGTQLQELYITPSMLNSNKWDTLANAAKWS
consensus         481 *.* * .*  .*.. *.  ***** **************.** .*  . ** ** ** **

A. hydrophila     525 QQNQAVLFDSHWIGGDPTLLAVYGWAAWSPSRAFITLRNPSNKPQTFLLDPQRQLEIPAG
Y. frederiksenii  527 RENSDVLVDTHWIGGDPTALDIYGWASWSKDKAIFGLRNPSDKEQSYYLDLTKDFEIPSG
consensus         541   *  ** *.******** * .**** **  .*   ***** * *.. **  .  *** *

A. hydrophila     585 EATRFTVQPRYGSNASVPATLDAPRLITLAPLELITLELTPAGTKPAAAEKS
Y. frederiksenii  587 QATSFTLKTVYGTNPSLAKDYSKPVIITLKPLETLVIEAIPVASTQ------
consensus         601  ** **.   **.* *.      * .*** *** . .*  *  ..  ......
```

Supplementary Figure S2: Phenotype of larvae following YacT injection. The arrows indicate arees of discolorations.

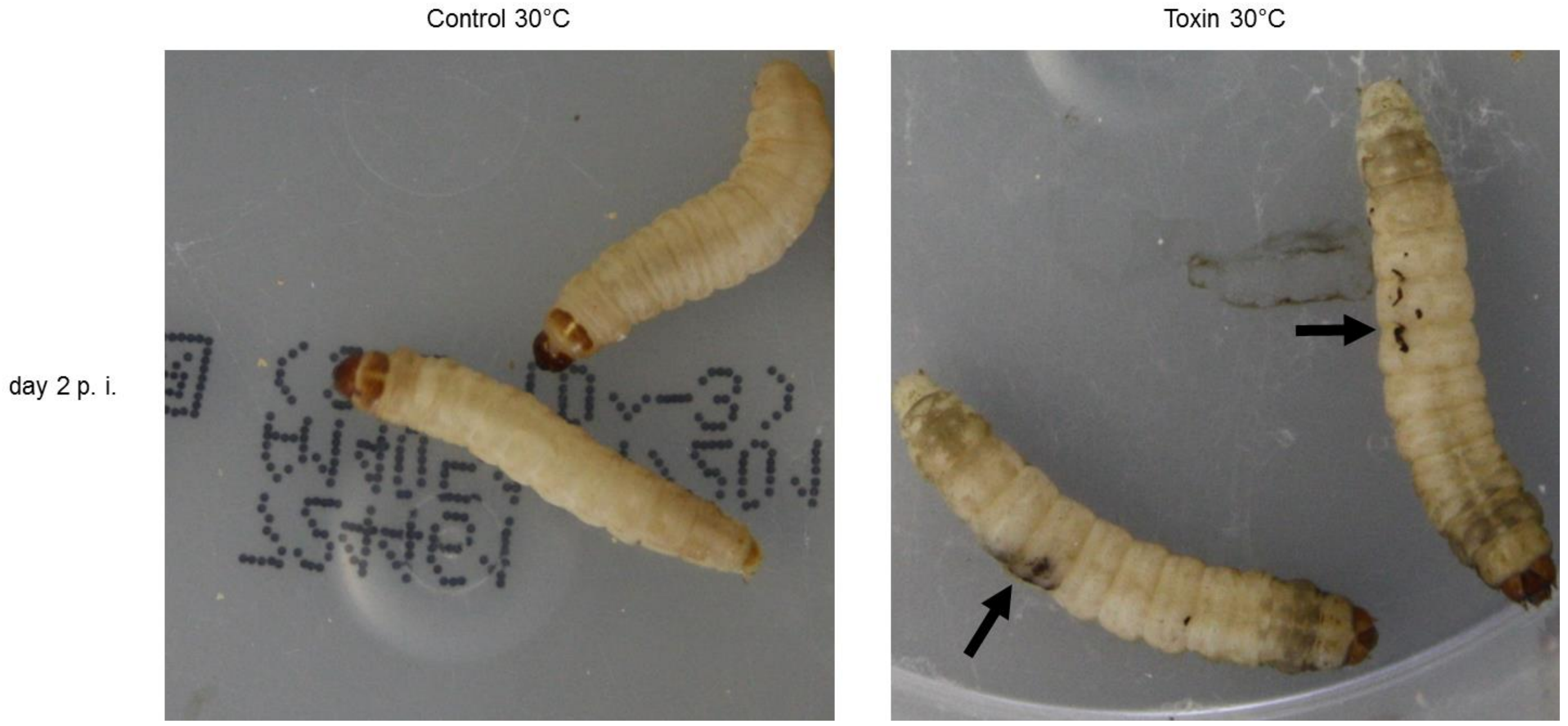

Supplementary Figure S3: SDS-PAA of purified YacT.

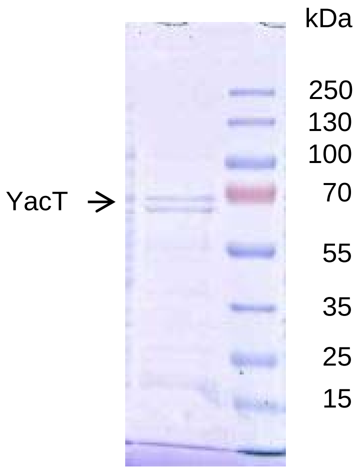

Supplement: Supplementary file 2 [file Data_Sheet_2.PDF]
